# 

**Published:** 1887-10

**Authors:** 


### BOOK NOTICES.

The Century—This monthly has come to the front and taken the lead of American magazines devoted exclusively to general literature, Its war articles, for which it has become justly celebrated, continue to be a main feature of its well stored columns, whilst the Lincoln papers, embracing a comprehensive history of Abraham Lincoln and his times, are progressing with unusual interest. Take it altogether, the *Century* may be regarded as the most useful as well as entertaining of our periodical visitors. At any rate, we are able to get more good reading matter out of it, with fuller illustrations, done to the highest touch of art, than any other magazine of its class is able to give us.

Alotypes, or Stenotypography, A System of Condensed Printing, etc., is the title of a pamphlet of ninety-two pages, by Henry H. Brown, a prominent lawyer of Battle Creek, Mich.

The object of the author is to present a new and simple method of writing and printing the English language, thus saving time and expense, and economizing materials, by reducing the size of volumes by making a single character perform the office of several of the letters now employed. The author illustrates his position by referring to the efforts of the *London Times*, to adopt the American method of spelling a number of words wherein the *u* is omitted from the final syllable, such as *favor*, parlor, etc., at a saving in labor and space, equal to $2,500 per annum, but was afterward abandoned out of regard to English prejudice of immemorial custom, for Noah Webster *wasn't* English, you know.

Mr. Brown offers to send this little work to any one remitting him four cents in stamps.

Horticultural Art Journal.—This time it is pœonias, white and red, as large as life, beautifully colored and shaded. There are also a number of other colored illustrations, any one of which is worth the price of this excellent floral monthly. Published by Stecher Lithographic Co., Rochester, N. Y.

Earnest Words is the title of a new monthly magazine devoted to the herculean task of destroying ignorance. This first number is replete of terse articles against some of the more prominent evils which beset the social state Knowing, as we do, personally, its editors, we feel assured that this publication will prove a powerful adjunct to the reform movement. It carries with its objects, our best wishes.

Published at 1398 Broadway, New York City.

